# Flexible Quantum-Dot Color-Conversion Layer Based on Microfluidics for Full-Color Micro-LEDs

**DOI:** 10.3390/mi13030448

**Published:** 2022-03-16

**Authors:** Panyuan Li, Jin Tao, Yongzhou Zhao, Yifang Sun, Kaili Fan, Licai Zhu, Wenchao Sun, Jinguang Lv, Yuxin Qin, Qiang Wang, Qinghui Zeng, Weibiao Wang, Shurong Wang, Jingqiu Liang

**Affiliations:** 1 State Key Laboratory of Applied Optics, Changchun Institute of Optics, Fine Mechanics and Physics, Chinese Academy of Sciences, Changchun 130033, China; lipanyuan_x@163.com (P.L.); zhaoyongzhou17@mails.ucas.edu.cn (Y.Z.); 13526696491@163.com (K.F.); zlc15254865629@163.com (L.Z.); sunwenchao20@mails.ucas.ac.cn (W.S.); jinguanglv@163.com (J.L.); qinyuxindavid@163.com (Y.Q.); wangqiang@ciomp.ac.cn (Q.W.); 2 University of Chinese Academy of Sciences, Beijing 100039, China; sunyifang18@mails.ucas.ac.cn; 3 State Key Laboratory of Luminescence and Applications, Changchun Institute of Optics, Fine Mechanics and Physics, Chinese Academy of Sciences, Changchun 130033, China; qhzeng@ciomp.ac.cn; 4 Space Optics Research Department I, Changchun Institute of Optics, Fine Mechanics and Physics, Chinese Academy of Sciences, Changchun 130033, China; wangwb@ciomp.ac.cn (W.W.); srwang@ciomp.ac.cn (S.W.)

**Keywords:** LED, flexible color-conversion layer, microfluidics, quantum dot

## Abstract

In this article, red and green perovskite quantum dots are incorporated into the pixels of a flexible color-conversion layer assembly using microfluidics. The flexible color-conversion layer is then integrated with a blue micro-LED to realize a full-color display with a pixel pitch of 200 μm. Perovskite quantum dots feature a high quantum yield, a tunable wavelength, and high stability. The flexible color-conversion layer using perovskite quantum dots shows good luminous and display performance under different bending conditions; is easy to manufacture, economical, and applicable; and has important potential applications in the development of flexible micro-displays.

## 1. Introduction

In recent years, micro-LEDs have been widely used in micro-displays [1,2,3], flexible displays [4,5], visible light communication [6,7,8,9], and optogenetics [10,11]. This is due to their unique advantages, including high brightness, high resolution, low power consumption, and rapid response [12,13]. Full-color micro-LED display has attracted a lot of research attention. However, it remains difficult to produce high-quality, full-color chips. A range of commonly accepted methods has been widely used to realize full-color micro-LEDs, including growth, transfer printing, and color-conversion techniques based on quantum dots.

In growth technology, high-resolution full-color micro-LEDs are realized by using the structure of nano-LEDs and multi-quantum wells to control color conversion. However, there are problems such as high production costs, low quantum efficiency, and color drift [14,15,16]. In transfer-printing technology, large-area, high-precision LED micro-particle transfer is carried out by adsorption microstructures, which include elastomer stamps, laser-induced transfer, electrostatic transfer, and roll-to-roll imprinting transfer [17,18,19]. However, as the size of the micro-LED decreases, some processes still require improvement, such as the precision of transfer equipment and the matching degree of transfer microstructures. In color-conversion technology, the full-color micro-LED structure is composed of blue/violet excitation light sources, as well as red and green color-conversion pixels. The color-conversion layers are generally produced by inkjet printing and photolithography [20,21]. Quantum dots have become popular materials because of their excellent performance in color conversion. In inkjet printing, nozzles are used to spray atomized quantum-dot materials [22,23,24]. In photolithography, photoresist and quantum dots are mixed to produce a color-conversion film in multiple photolithography [25,26]. Inkjet printing is suitable for the production of larger pixels, because pixel size is limited by the nozzle. The fabrication of the photolithographic method is simple, and the size and morphology of the quantum-dot pixel can be precisely controlled. However, the stability and color-conversion efficiency of the quantum dot after doping with photoresist still needs improvement. Microfluidic technology can accurately manipulate fluids in nanoscale spaces and is widely used in chemistry, biology, and medicine [27,28]. The micron-sized microchannels are suitable for making color-conversion layers of micro-LEDs.

On the other hand, flexible micro-LED devices have become a research hotspot in the fields of wearable devices, augmented reality, biomedicine, and vehicle displays due to their mechanical properties that allow twisting, folding, and stretching. Over the past few years, researchers have fabricated flexible blue or ultraviolet micro-LED devices using techniques such as nanowire growth, mass transfer, and metal bonding [29,30]. However, due to the difficulty of manufacturing full-color flexible displays, there are few technical methods to develop full-color flexible micro-LEDs. It is necessary to integrate a flexible quantum-dot color-conversion layer based on the current mature flexible GaN-based micro-LED and study the fabrication of flexible full-color micro-LED devices. In this paper, we propose a technical method for preparing a flexible quantum-dot color-conversion layer (FQCL) to achieve a full-color flexible display using microfluidics. The pixel size of 200 μm includes RGB subpixels. The photoluminescence (PL) intensity changes of red and green FQCL subpixels are 26% and 3%, respectively, when the bending radius is 15 mm. The PL intensity changes of red and green FQCL subpixels are 29% and 5%, respectively, after bending 1000 times. The change of the color gamut is less than 4% after bending 1000 times.

## 2. Materials and Methods

**Figure 1:**
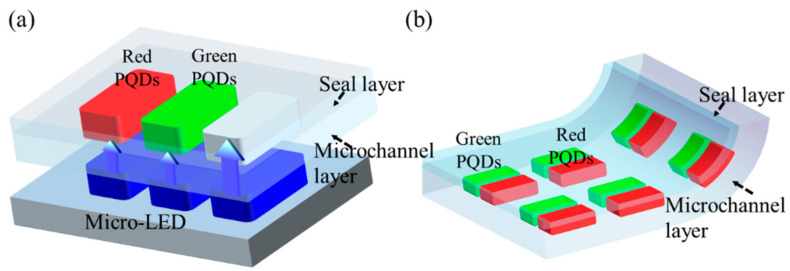
Schematic diagram of (**a**) a full-color micro-LED array display device and (**b**) the bending of the FQCL.

A full-color display is usually composed of red, green, and blue pixels. It uses spatial color mixing to produce a color image by adjusting pixel drive voltage at a high level of precision. Figure 1a presents a schematic diagram of a full-color micro-LED display device. The device consists of a blue micro-LED array at the bottom and a flexible quantum-dot color-conversion layer at the top. The blue micro-LED array is used as the blue light source and as the excitation light source for quantum dots. For the other two sources of full-color display, red and green perovskite quantum dots (PQDs) in the pixels of the FQCL emit red and green light after being illuminated by the blue light.

The fabrication of FQCL includes making SU8 molds, making a flexible microchannel layer and seal layer, flexible chip bonding, and injecting the quantum-dot solution. Both the microchannel layer and the seal layer of the chip are made of polydimethylsiloxane (PDMS) materials with good bending properties. A schematic diagram of the bending of the FQCL chip is shown in Figure 1b.

**Figure 2:**
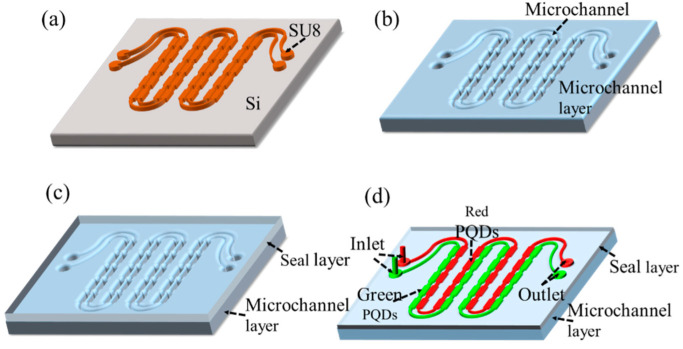
Schematic diagram of the production process of FQCL chip: (**a**) SU8 silicon mold, (**b**) flexible microchannel layer, (**c**) flexible PDMS chip, and (**d**) FQCL.

The SU8 molds were made using photolithography on a polished silicon substrate, as shown in Figure 2a. First, the silicon wafer was cleaned with a piranha solution (H_2_SO_4_:H_2_O_2_:H_2_O:5:1:1), and the surface characteristics were modified with oxygen plasma (120 W, O_2_ 200 sccm, 60 s). The SU8 photoresist (500 rpm 18 s, 1000 rpm 30 s) was rotated on the silicon wafer, and the solvent in the photoresist was evaporated by soft drying (95 °C for 5 min). The silicon wafer was exposed under ultraviolet light and developed by impregnation in propylene glycol methyl ether acetate. Finally, the silicon wafer was baked at 150 °C for 5 min to remove the remaining solvent in the photoresist, enhance the adhesion of the photoresist to the silicon wafer, and ensure the quality of the pattern.

The fabrication of the flexible PDMS microchannel layer and the seal layer included preparing a PDMS solution, removing bubbles in the solution, and curing the PDMS. For this, first, the classic choice of PDMS for microfluidics is Dow Corning Sylgard 184, a two-part prepolymer with a mix mass ratio of siloxane and cross-linker/curing agent of 10:1. It was stirred with a glass stick to mix the siloxane and cross-linker and to generate bubbles. Then, the thoroughly stirred PDMS solution was placed in a low-pressure vacuum oven for 30 min to remove the air bubbles. Finally, the PDMS solution was poured into the molds of the microchannel layer and the seal layer and placed in the oven. The flexible microchannel layer was baked at 65 °C for 20 min, and the flexible seal layer was baked at 65 °C for 15 min to retain viscosity. Figure 2b shows the schematic diagram of the flexible PDMS runner layer after curing.

The chip bonding was realized by thermocompression bonding. After curing, the flexible microchannel layer and the flexible sealing layer were stripped with a dicing knife, and the liquid inlet and outlet of the microchannel layer were drilled. To increase the success rate, the flexible microchannel layer and the seal layer were cleaned with oxygen plasma (120 W, O_2_ 200 sccm, 60 s). The two PDMS pieces were aligned and bonded under the microscope and then baked in an oven to complete the bonding (80 °C for 3 h). The effect of the chip was observed under a microscope, and the chip with complete lamination, as well as intact microchannels and liquid ports, was selected. Figure 2c shows a schematic diagram of a flexible color-conversion layer chip after bonding.

The PQDs were dissolved in n-hexane, and the red and green quantum-dot solutions were injected into the two rows of microchannels by liquid injection to make an FQCL, as shown in Figure 2d.

## 3. Results and Discussion

### 3.1. Preparation and Test of Perovskite Quantum Dots

**Figure 3:**
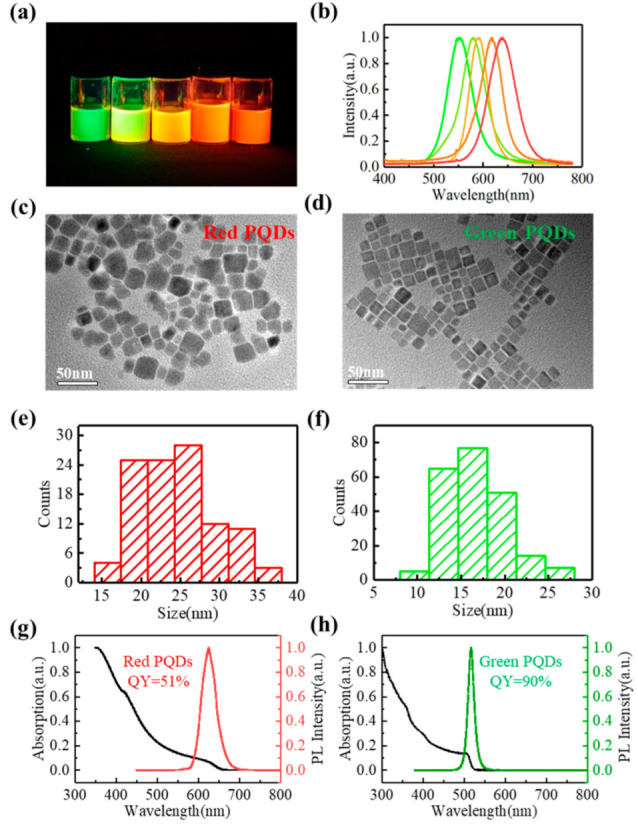
The (**a**) solution and (**b**) emission lines of different color PQDs. TEM image of (**c**) red and (**d**) green PQDs. Particle size distribution of (**e**) red and **(f**) green PQDs. Spectral curves of (**g**) red and (**h**) green PQDs.

Due to their superior optical properties, such as a wider color gamut, narrower emission spectrum, and adjustable emission wavelength, PQDs have been more broadly researched and applied in optoelectronic devices than traditional quantum dots [31,32,33,34]. In this work, as mentioned, CsPbX_3_ (X = Cl, Br, I) quantum dots were prepared by thermal injection at high temperatures [35]. PQDs with different emission wavelengths were obtained by accurately controlling the ratio of halide atoms and the technical parameters of the thermal injection. The quantum-dot solution and its emission spectrum curves at some wavelengths are shown in Figure 3a,b. In this study, green (CsPbBr_3_) and red (CsPbI_2_Br) PQDs are the main luminescent materials used to manufacture FQCLs.

We purchased materials from Sigma-Aldrich and prepared the perovskite quantum dots. First, we prepared the Cesium Oleate Precursors. Cesium carbonate (Cs_2_CO_3_, 1.2 mmol), oleic acid (OA, 4 mmol), and octadecene (ODE) were added into a 50 mL three-necked flask, which was heated to 120 °C. The contents were stirred for 60 min under an argon atmosphere. A colorless and transparent cesium oleate precursor solution was obtained after 30 min reaction at 160 °C. This was followed by the synthesis of CsPbX (X=Cl, Br, or I) PQDs. Next, 0.4 mmol PbX_2_ (0.149 g PbBr_2_ for green PQDs, 0.0489 g PbBr_2_, and 0.125 g PbI_2_ for red PQDs) and 24 mL ODE solvent were added to the three-necked flask. The solution was stirred for 30 min in an argon flow environment and then heated to 120 °C for 30 min. The mixture of OA (1 mL) and oleamine (OAm, 3 mL) was injected into a reaction flask using a syringe. After it was stirred for a few seconds, PbX_2_ was completely dissolved into a transparent solution. It was then heated to 180 °C, and the cesium oleate (2 mL) precursor solution was quickly injected. After the designed reaction time, the growth of nanocrystals was terminated, and the reaction mixture was cooled in an ice-water bath. Finally, the sample was obtained by adding ~30 mL toluene at 9000 rpm for 10 min followed by centrifugation. After centrifugation, the supernatant was poured out, and the nanocrystals at the bottom of the centrifuge tube were redispersed in n-hexane. After being purified twice, the obtained PQDs were dispersed in n-hexane.

The main chemical composition of the green PQDs was CsPbBr_3_, and the composition of the red PQDs was CsPbI_2_Br. The red and green PQDs were assessed with a transmission electron microscope (TEM). The PQDs solution was ultrasonically shaken, dropped on carbon film, and dried. In Figure 3c,d, the CsPbBr_3_ and CsPbI_2_Br PQDs present a cubic shape. Statistical analyses showed that the sizes of green PQDs were about 16.68 ± 3.5 nm, and the sizes of redPQDs were about 24.51 ± 4.8 nm, as shown in Figure 3e,f. To verify the reliability of the green and red PQDs in color conversion, we tested the ultraviolet-visible absorption spectrum and the PL spectrum of the PQDs, as shown in Figure 3g,h. PQDs were dispersed in n-hexane, and the quantum-dot solution was excited by ultraviolet light at 395 nm. The green and red emissions peaks were at 514 nm and 625 nm, and the full width at half-maximum (FWHM) values was 21 nm and 40 nm, respectively. The quantum yield (QY) of the green PQDs was 90%, and the QY of the red PQDs was 51%.

### 3.2. Optical Image of FQCL

**Figure 4:**
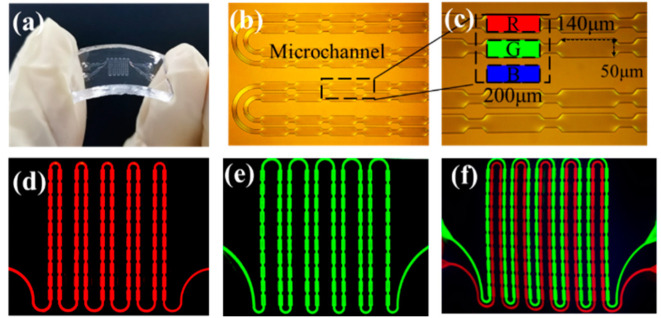
(**a**) Image of a physical flexible chip bending. (**b**) Flexible chip microchannels and (**c**) partially enlarged view of microchannels. (**d**) Red pixel array, (**e**) green pixel array, and (**f**) two-color pixel array.

After bonding, the flexible color-conversion layer is shown in Figure 4a. In Figure 4b,c, the microchannels are displayed, as well as a partially enlarged view of the microchannels. The number of pixels in the flexible color-conversion layer is 10 × 10, the monochromatic pixels are 140 × 50 μm, the full-color pixels are 140 × 182 μm, the pitch of the full-color pixels is 200 μm, and the depth of the SU8 microchannels is 30 μm. Because the red and green microchannels are separated, red and green PQDs can be injected simultaneously or separately. As shown in Figure 4d–f, the excitation light is filtered, and the red pixel array, the green pixel array, and the two-color pixel array were observed under the microscope. Using photolithography, the fabrication of an FQCL with microfluidics reduces the amount of quantum dots, because the quantum dots only flow in microchannels. For a typical full-color display with a resolution of 1920 × 1080, the dosage of red and green PQDs is ~2 mg, and the concentration of the PQD solution used is 2 mg/mL. The length, width, and height of the microchannel pixels can be precisely adjusted during the mold-making process so we can choose the pixel size that is most appropriate for achieving the best display performance.

### 3.3. Bending Test of FQCL Chips

The mechanical adaptability of flexible optoelectronic devices is important for measuring their service life, and the bending radius, as well as performance changes after bending, are essential indicators of mechanical adaptability.

**Figure 5:**
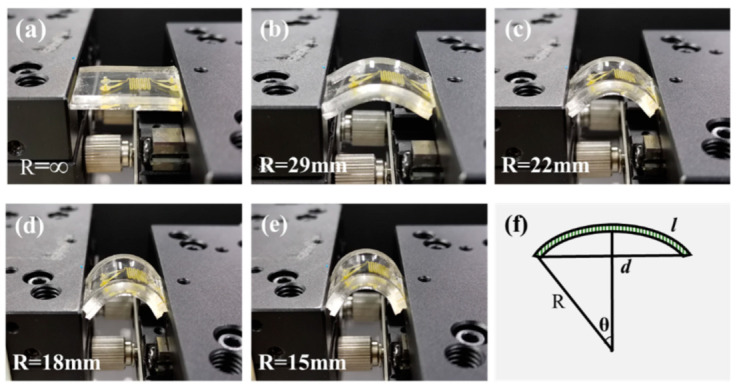
(**a**–**e**) Flexible chips with different bending radii, and (**f**) the schematic diagram of a bent FQCL.

To verify the reliability of the FQCL chips, their bending-state performance was tested. The FQCL was fixed with a precision displacement platform and bent under different curvature radii, as shown in Figure 5. The length (*l*) of the FQCL was fixed. The bending radii (*R*) of the FQCL were infinity, 29, 22, 18, and 15 mm, respectively. During the experiment, the corresponding radius was achieved by adjusting the chord length (*d*). According to the chord length formula and the trigonometric function formula, the relationship between *d*, *l*, and *R* is expressed by Equation (1), as shown in Figure 5f.

(1) $$d=2Rsin\left(\frac{l}{2\pi R}\;\pi \right)$$

**Figure 6:**
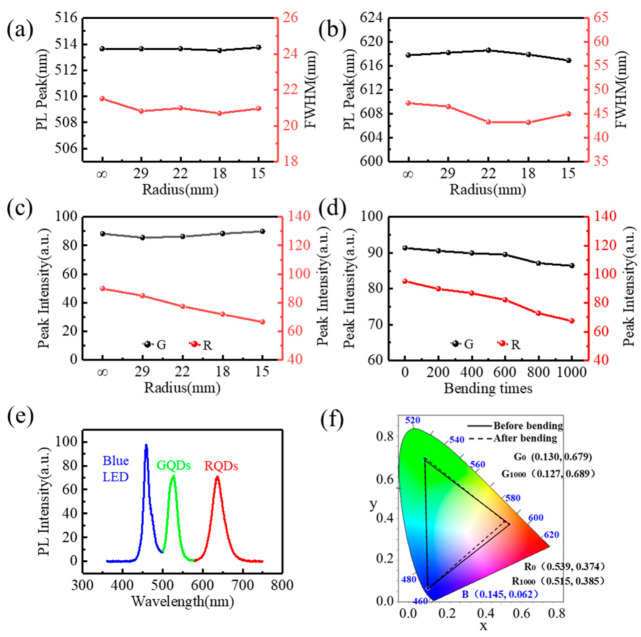
Central wavelength and FWHM variation curves at different bending radii of (**a**) green FQCL and (**b**) red FQCL. (**c**) Peak intensity variation curves of green and red FQCL at different bending radii. (**d**) Peak intensity variation curves at different bending times of green and red FQCL. (**e**) Full-spectrum curve of red, green, and blue. (**f**) Color gamut diagram.

To verify the display performance of the FQCL chips under different bending conditions, the luminescence characteristics at different bending radii and bending times were tested, as shown in Figure 6.

Under the excitation of a 460 nm blue LED, spectrometers (Ocean Optics USB2000+, Ocean Insight, Rochester, NY, USA) were used to test the PL spectra of the red and green FQCL subpixels, fixed on different columns, with bending radii of infinity, 29, 22, 18, and 15 mm. Figure 6a,b show the emission peak and FWHM curves of the green and red FQCLs under different bending radii, respectively. As the bending radius of the chip decreases, the emission peak of the green FQCL fluctuated around 514 nm, with a variation range of less than 0.05%, and the FWHM fluctuated around 21 nm, with a variation range of less than 2.5%. The emission peak of the red FQCL fluctuated around 618.5 nm, and the variation range was less than 0.5%. The FWHM is about 45 nm, and the fluctuation range was less than 10%. As the bending radius fell, the naturalized relative peak intensity of the green FQCL emission spectrum was about 88, with a fluctuation range of less than 3%. The naturalized relative peak intensity of the red FQCL showed a gradual decline of about 26%, as shown in Figure 6c. Under different bending radii, the emission peak, FWHM, and peak intensity of the green FQCL emission spectrum were relatively stable. According to theory and experiments, we know that the shape of CsPbBr_3_ PQDs are cube, while that of CsPbI_2_Br PQDs are rectangle. Compared with CsPbBr_3_ PQDs, CsPbI_2_Br PQDs have a larger size and size range. Based on these findings, we inferred that when the bending radius increased, and under the action of stress, this change of the PQDs arrangement led to larger gaps between the PQDs, as well as a decrease in the absorption rate and conversion efficiency of the PQDs film. Compared with the green PQDs, the size and gaps of the red PQDs are larger, so the density of the film decreased more obviously in the bending state, and the detected emission intensity decreased significantly.

To verify the flexural resistance of the FQCL, the two ends of the chip were fixed on the clamps of the electronically controlled displacement platform (Zolix SC300-2B, Zolix, Beijing, China). The PL intensity of the chip after repeated bending was detected, and the bending range was found to be 0–1000 times. In Figure 6d, with an increase in bending times, the naturalized relative peak intensity of the green FQCL emission spectrum decreased, but it maintained good luminescence performance, with a less than 5% decrease. The peak intensity of the red FQCL also decreased with increased bending times and had a decrease of about 29%. After many bends, the red luminous intensity decreased significantly. This phenomenon is similar to the data presented in Figure 6c. The stress caused by bending affected the spectral absorption efficiency and energy conversion efficiency of PQDs. Repeated bending also changed the density of the PQDs film, so the relative intensity of the PQDs decreased with a decrease in density. Because of the larger size and shape, the relative intensity of the red PQDs decreased more obviously than that of the green PQDs.

The color gamut is an important indicator of a display device, and it reflects the richness of the colors that the display can show. As an important material that can create a wide color gamut, PQDs are widely used in LED displays and solid-state lighting. Figure 6e shows the full spectrum curve based on blue LEDs, as well as red and green PQDs. Figure 6f shows the color gamut of blue LEDs, as well as red and green PQDs, both without bending and with bending at 1000 times. After bending, the color coordinates of the red and green PQDs shifted slightly, but the change in the color space coverage was less than 4%, which could better meet the requirements of full-color display devices.

### 3.4. Temperature Test of FQCL Chips

**Figure 7:**
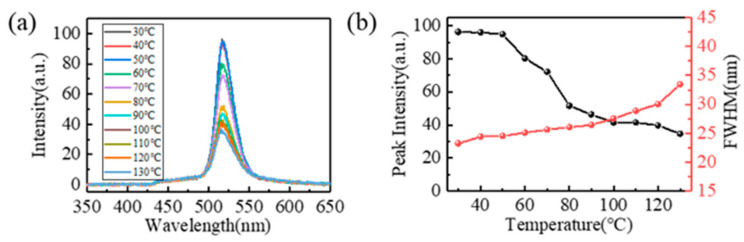
Luminescence performance test of FQCL at different temperatures. (**a**) Spectral curves of FQCL, (**b**) peak intensity, and FWHM of FQCL.

As the LED functions, part of the input electric energy is converted into heat through non-radiative recombination, which increases the temperature of the LED surface. However, the fluorescent intensity of the quantum dots decreases as the temperature increases. If the temperature does not change much, it can be controlled by the physical process of thermal activation, and this thermal-activation process is reversible. The FQCL chip made with microfluidics can disperse the heat generated by the LED. The luminescence of the FQCL was tested at 30–130 °C, as shown in Figure 7. The relative peak intensity of FQCL pixels at 70 °C exceeded 70% of the peak intensity at room temperature. For comparison, R-S Liu reported [36] that the relative peak intensity of untreated CsPbBr_3_ dropped to 25% at 75 °C. This device shows obvious advantages in improving the thermal stability of PQDs.

FQCLs were fabricated using microfluidics. If combined with flexible blue micro-LEDs, flexible full-color micro-LED devices could be fabricated without mass transfer. The process is simple and easy to operate. Of course, the distance and geometrical structure between the FQCL and the blue micro-LED may cause blue light leakage and color crosstalk problems. However, the shape and size of the FQCL pixels, based on microfluidics, are easier to adjust and manufacture. Meanwhile, it is also possible to adjust the angle of light divergence by designing the seal layer as a micro-lens array to improve the light-emitting performance of the device. These problems and solutions will be investigated in future work.

## 4. Conclusions

In this study, FQCLs were fabricated using microfluidics. Red and green PQDs with high luminous efficiency were synthesized using thermal injection, and the QY of the red and green PQDs reached 51% and 90%, respectively. The luminescence performance of the FQCL was tested at different bending radii, and again after multiple bends. The two-color FQCL showed good display performance under different bending conditions. As the bending radius decreased, the naturalized relative peak intensity of the green FQCL emission spectrum fluctuated less than 3%, while the relative peak intensity of the red emission spectrum decreased with an amplitude of about 26%. The PL intensity changes of the red and green FQCL were 29% and 5%, respectively, after bending 1000 times. The color space coverage of the blue LED, as well as the red and the green PQDs, after bending 1000 times, only had a 4% drift. This still meets the requirements of full-color displays. This method does not require mass transfer and has the advantages of high precision, controllable size and shape, and economy. It can promote the industrialization and commercialization of flexible micro-displays, and has considerable potential applications, such as for wearable devices, vehicle displays, and electronic skin.

## Data Availability

This study did not report any data.
